# Real-World Use and Adverse Events of SARS-CoV-2 Vaccination in Greek Patients with Inflammatory Bowel Disease

**DOI:** 10.3390/jcm11030641

**Published:** 2022-01-27

**Authors:** Eleni Orfanoudaki, Eirini Zacharopoulou, Vassiliki Kitsou, Konstantinos Karmiris, Angeliki Theodoropoulou, Gerassimos J. Mantzaris, Maria Tzouvala, Spyridon Michopoulos, Evanthia Zampeli, Georgios Michalopoulos, Pantelis Karatzas, Nikos Viazis, Christos Liatsos, Giorgos Bamias, Ioannis E. Koutroubakis, on behalf of the Hellenic Group for the Study of IBD

**Affiliations:** 1 Department of Gastroenterology, University Hospital of Heraklion, Medical School, University of Crete, 71110 Heraklion, Greece; ikoutroubakis@gmail.com; 2 Department of Gastroenterology, General Hospital of Nikaia Piraeus “Ag. Panteleimon”-General Hospital Dytikis Attikis “Agia Varvara”, 12351 Athens, Greece; eirinizachar@gmail.com (E.Z.); tzouvalam@gmail.com (M.T.); 3 Gastroenterology Unit, 3rd Academic Department of Internal Medicine, National and Kapodistrian Univeristy of Athens, “Sotiria” General Hospital, 11527 Athens, Greece; vassosgkp@gmail.com (V.K.); gbamias@gmail.com (G.B.); 4 Department of Gastroenterology, Venizelio General Hospital, 71409 Heraklion, Greece; kkarmiris@gmail.com (K.K.); angelikitheodoropoulou@yahoo.co.uk (A.T.); 5 Department of Gastroenterology, General Hospital of Athens, Evaggelismos-Polykliniki, 10676 Athens, Greece; gjmantzaris@gmail.com (G.J.M.); nikos.viazis@gmail.com (N.V.); 6 Department of Gastroenterology, General Hospital of Athens “Alexandra”, 11528 Athens, Greece; michosp5@gmail.com (S.M.); evazamb@gmail.com (E.Z.); 7 Department of Gastroenterology, General Hospital of Piraeus “Tzaneio”, Piraeus, 18536 Athens, Greece; gmicha78@hotmail.com; 8 Department of Gastroenterology, National and Kapodistrian University of Athens, General Hospital of Athens “Laiko”, 11527 Athens, Greece; panteliskaratzas@gmail.com; 9 Gastroenterology Department, 401 General Army Hospital of Athens, 11525 Athens, Greece; cliatsos@yahoo.com

**Keywords:** COVID-19, Crohn’s disease, vaccine, ulcerative colitis

## Abstract

Since inflammatory bowel disease (IBD) patients were excluded from vaccine authorization studies, limited knowledge exists regarding perceptions and unfavorable effects of COVID-19 vaccination in this group. We aimed to investigate the real-world use and adverse events (AEs) of COVID-19 vaccines in Greek IBD patients. Fully vaccinated IBD patients followed in Greek centers were invited to participate. All patients filled out an anonymous online survey concerning the vaccination program, which included information regarding demographics, clinical characteristics, treatment, vaccination perceptions and potential AEs. Overall, 1007 IBD patients were included. Vaccine hesitancy was reported by 49%. Total AEs to vaccination were reported by 81% after dose 1 (D1) and 76% after dose 2 (D2), including isolated injection site reactions (36% and 24% respectively). Systemic AEs were more common after D2 (51%, D2 vs. 44%, D1, *p* < 0.0001). Very few patients reported new onset abdominal symptoms (abdominal pain 4% (D1), 6% (D2) and diarrhea 5% (D1), 7% (D2)). There were no serious AEs leading to emergency room visit or hospitalization. In multivariate analysis, AEs occurrence was positively associated with young age and female gender (*p* < 0.0005 for both doses), whereas inactive disease was negatively associated with AE in D1 (*p* = 0.044). SARS-CoV-2 vaccination in Greek IBD patients demonstrated a favorable and reassuring safety profile.

## 1. Introduction

The severe acute respiratory syndrome Coronavirus-2 (SARS-CoV-2) was first reported at Wuhan, China in late 2019. Shortly after, it became apparent that it was a highly contagious virus spreading rapidly, alerting the majority of health authorities worldwide. Therefore, the World Health Organization declared the coronavirus associated disease (COVID-19) a pandemic in March 2020. COVID-19 is characterized by severe illness and high mortality, which has raised concerns in the medical community and accelerated vaccination procedures, as the only effective and rapid intervention with which to confront the pandemic.

Since December 2020, when vaccines against COVID-19 became available, a growing number of doses have been administered worldwide. Priority was given in most countries in specific vulnerable populations, such as those under immunosuppressive treatment, considering their susceptibility to infectious complications. For the time being, four vaccines against COVID-19 have received emergency use authorization (EUA) in Europe. They have been manufactured either with the mRNA or the viral vector vaccine technologies that are theoretically safe for immunosuppressed patients.

Inflammatory bowel diseases (IBD), including ulcerative colitis (UC) and Crohn’s disease (CD), belong to the group of immune-mediated diseases. Treatment options include immunosuppressive drugs such as corticosteroids, conventional (azathioprine, methotrexate) and newer synthetic disease modifiers (tofacitinib), as well as biologic agents (anti-TNFa, anti- α4β7 integrin, anti-IL12/23) [1]. Live attenuated vaccines are contraindicated for immunosuppressed IBD patients [2]. All the aforementioned medications, excluding, probably, the gut selective anti–α4β7 integrin, could potentially jeopardize the immune response to SARS-CoV-2 vaccination. The majority of studies on vaccination against H1N1 viral infection have shown reduced seroconversion rates in IBD patients under immunosuppressive therapy compared with the general population [3]. Similar experience, with suboptimal vaccine response, also exists for the pneumoniococcus, hepatitis B and varicella zoster vaccines [3].

Although IBD patients on all sorts of immunosuppressive agents were excluded from clinical trials preceding vaccine EUA [4,5,6], emerging data suggest that they—even those under immunosuppressants—are not at higher risk of developing adverse events (AEs) after COVID-19 vaccination, when compared with control cohorts [7,8,9]. This applies both to mild and severe AEs or AEs of special interest, both topical and systemic. However, in certain reports, biologic agents in general or anti-TNFα agents in particular turned out to be either risk [10] or protective factors [7] for AE occurrence. Young age, female gender, vaccine type and prior COVID infection have all been associated with AEs in healthy controls as well as in the IBD subpopulation [7,10]. Regarding a possible impact of vaccines on disease activity, no correlation has been found as assessed by steroid need or changes in clinical disease activity scores [8,9].

The aim of this study was to expand these preliminary observations by using an electronic survey in order to investigate both the implementation of the vaccination program against COVID-19 in Greek IBD patients and any potential AEs following vaccination.

## 2. Materials and Methods

### 2.1. Study Design and Population

Adult IBD patients followed in Greek centers, (secondary and tertiary care hospitals, as well as private practices) were invited to fill an anonymous online survey between 16th April and 15th September 2021 concerning the vaccination program. The invitation as well as reminder requests were sent via electronic mail either by the treating physicians or the Hellenic Association of patients with Crohn’s and Colitis (HELLESCC). Relevant information and call for completing the survey were also posted in the HELLESCC website (http://www.crohnhellas.gr/ accessed on 15 September 2021). Patients were requested to fill the survey after they had completed the vaccination program. The survey consisted of a questionnaire with data regarding patients’ demographics, clinical status, treatment, vaccination perceptions and potential AEs after the first (D1) and the second doses (D2).

Treatment was classified into five categories: biologic monotherapy, combination of a biologic with conventional immunomodulators (IMM), IMM monotherapy, corticosteroids and no immunomodifiers. Biologics included anti-tumor necrosis factor (TNF)-α, anti-α4β7 integrin and anti-interleukin (IL)-12/23 agents whereas IMM azathioprine and methotrexate. Analysis included these five categories but also all different regimens separately.

During this time period, four vaccines were granted a conditional (emergency) marketing authorization for COVID 19. These included messenger–RNA vaccines (BNT162b2 (Pfizer-BioNTech) and mRNA-1273 (Moderna/NIH)) and adenovirus viral vector vaccines (ChAdOx1 (Astra Zeneca) and Ad26.CoV2.S (Johnson & Johnson)). All vaccines require two doses of the same type of vaccine administered several weeks apart, except for Ad26.CoV2.S (Johnson & Johnson), which requires only one dose.

AEs were initially stated as any kind of new symptom or sign onset that were further divided to localized (at the injection site) or systematic. The latter involved fatigue, headache, allergic reactions, fever, lymphadenopathy, myalgias/arthralgias and gastrointestinal disorders (diarrhea, abdominal pain, loss of blood in stool). All the aforementioned AEs were stated as predefined options to choose, but free text response options were offered as well. AEs were studied as a whole and separately. Patients receiving the monodosic vaccine J&J replied to the questionnaire regarding only D1 AEs.

The study was conducted under the auspices of the Hellenic Study Group of Idiopathic Inflammatory Bowel Diseases (EOMIFNE). The protocol has been approved by the ethical committees of all participating hospitals according to national legislation, and informed consent was obtained in all cases.

### 2.2. Statistical Analysis

Mean (standard deviation, SD) or median (interquartile range, IQR) values were calculated according to data distribution (normal or not). Comparison between AEs prevalence in D1 and D2 AEs was performed using Wilcoxon test for paired samples. Presence of AEs after both vaccination doses as a dependent variable was correlated with demographics, disease characteristics and treatment using univariate logistic regression analysis. To adjust for potential confounders, covariates with *p* values less than 0.1 in univariate analysis were tested in a multiple logistic regression analysis. The threshold for statistical significance was set at *p* < 0.05

## 3. Results

### 3.1. Baseline Characteristics

A total of 1128 IBD patients responded to the online survey. However, 121 responders were excluded for reporting incomplete data or duplicate responses. Thus, analysis of the results was based on complete data from 1007 (89.3%) responders. Fifty-one per cent were male. The median (IQR) age was 44 (35–55) and the median disease duration was 10 (5–16) years respectively. The majority were diagnosed with CD (64.3%) and 26 (2.6%) patients reported a history of COVID-19 infection. Detailed demographics and clinical characteristics of the study population are presented in Table 1.

**Figure 1:**
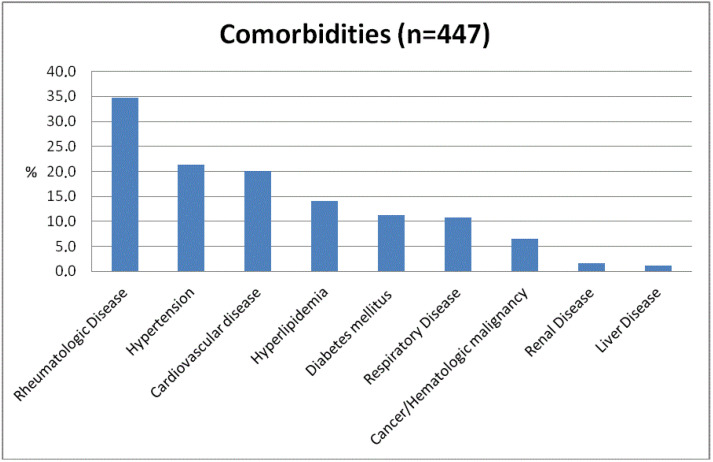
Prevalence of comorbidities (*N* = 447) of inflammatory bowel disease patients included in the study.

Overall, 447 of 1007 responders (44.4%) reported additional co-morbidities the majority been rheumatologic disease (35%), hypertension (21%), and hyperlipidemia (20%) (Figure 1).

The median (IQR) time between 2nd vaccine dose and questionnaire completion was 15 (5–43) days. During the vaccination period 213 (21.2%) patients did not receive any treatment or any conventional immune modifiers (only mesalazine and budesonide recipients were included). The rest were under treatment with biologic monotherapy (53.5%), biologics in combination with IMM (10.5%), IMM monotherapy (13%) or concomitant treatment with corticosteroids and IMM (3.2%).

### 3.2. Vaccination Perceptions

More than half of the patients (51%) stated that they felt confident with being vaccinated, whereas the remaining patients although reluctant initially, proceeded to vaccination, when they were convinced for its benefits. There was a remarkable m-RNA vaccine predominance of 10:1 ratio over viral vector vaccines.

### 3.3. Adverse Events

The overall prevalence of reported AEs was 81% and 76% after dose 1 (D1) and dose 2 (D2), respectively. Serious AEs leading to emergency room visit or hospitalization were not reported. Local symptoms or signs at injection site were reported by 73% after D1, and 67% after D2. The frequency of systemic AEs (including fatigue, allergy, fever, lymphadenopathy, joint/muscle pain or gastrointestinal symptoms) was 44% and 51% after D1 and D2, respectively. Increased rates of systemic AEs from D1 to D2 were statistically significant in all subcategories except for allergic reactions, whereas injection site symptoms showed a significant decrease from D1 to D2. Fatigue was the most commonly reported systemic AE followed by muscle/joint pain. The development of new abdominal symptoms potentially related to IBD included abdominal pain in 4% and 6%, as well as diarrhea in 5% and 7% of patients after D1 and D2, respectively. An overview of all recorded AEs is shown in Table 2.

In the univariate analysis, female gender and young age were significantly associated with AE occurrence after both doses (Table 3). mRNA vaccines were associated with fewer AEs after D1 and more AEs after D2. Patients with prior COVID-19 infection presented with more AEs after D2 (*p* = 0.027), whereas those with reported inactive disease developed fewer AEs after D1 (*p* = 0.012). Higher BMI was negatively associated with AEs only in D2 (*p* = 0.001). Regarding blood type, AB+ was positively correlated with development of AEs in both D1 and D2 (*p* = 0.033 and *p* = 0.028 respectively) (Table 4). No correlation between AE occurrence after either dose and presence of any kind of comorbidities was found (in comorbidity sub analysis only rheumatologic disease was found to be a risk factor for AEs after D2 (*p* = 0.003) which was not further confirmed in multivariate analysis).

The association of AE with young age and female gender remained significant in both vaccine doses after adjusting for confounding factors in the multivariate analysis (Table 5) (young age (*p* < 0.001 for D1 and *p* < 0.0001 for D2), female gender (*p* < 0.001 for D1 and *p* < 0.0001 for D2)). No association was found with the presence of comorbidities or relative medications (statins, etc.) nor with the use of IBD medications, including advanced therapy (*p* > 0.05) except from corticosteroid use in D2 (*p* = 0.03) but we did not proceed with sub-analysis for corticosteroid monotherapy or combination with biologics and/or IMMs due to small sample size. Blood type AB+ was independently associated with AEs after both doses. Inactive disease remained a negative risk factor after D1 as well.

## 4. Discussion

This study aimed at reporting the experience on the acceptance and safety of vaccines against COVID-19 in IBD patients. Interestingly, it was highlighted that the occurrence of AEs after vaccination against COVID-19 in Greek IBD patients was similar to that reported in other ethnic IBD and non-IBD populations [7,8,9,10,11,12]. No severe disease relapse or serious AEs including hospitalizations for any reason, severe allergic reactions, thromboembolic events or myopericarditis were recorded. Young age, female gender, blood type AB+, but not IBD-related medications, were found to be associated with the development of AEs in the multivariate regression analysis.

Female gender and young age have been identified in various studies as risk factors for AEs after vaccination not only against SARS-COV-2 [13,14] but also other infectious agents [15,16]. This female predominance could be explained by gender-related genetic factors as well as by the role of hormones, such as estradiol and testosterone, on the immune response and cytokine production after vaccination [16]. The same applies for age, as younger people appear to elicit a more potent immune response with increased production of CRP, IL-6, IL-10 and other cytokines that are consequently related with more AEs [17].

Regarding blood type, there is evidence that O and Rh-negative blood groups are associated with a lower risk for SARS-CoV-2 infection and severe COVID-19 disease [18]. B and AB blood groups have been found as negative predictors of early BNT 162b2 vaccine response [19]. A retrospective analysis of data from 2878 healthy volunteers, health care workers and students did not find any relationship between mRNA vaccine reactogenicity, as implied by side effects and ABO blood type [20]. The present study showed a significant positive association between blood type AB+ and AEs after COVID-19 vaccination in IBD patients. Whether this is a characteristic unique for IBD patients or also for healthy individuals and the possible impact it may have in clinical outcomes and in serologic response remains to be investigated by larger studies.

In agreement with previous studies, we showed that prior COVID-19 infection is correlated with the presence of AEs to vaccination [21,22]. Moreover, a statistically significant increase of systemic AEs after D2 compared to D1 was reported. Both findings can be interpreted by the induction of a pre-existing immunization process from prior COVID-19 infection and D1, respectively [21,22]. In keeping with this observation, there is evidence that these populations present with higher post vaccination antibody titers [22].

There is much concern that COVID-19 vaccines may trigger an IBD flare. Based on reassuring data elucidated from research on other vaccinations [23], members of the International Organization for the Study of IBD (IOIBD) in collaboration with expert immunologists and vaccinologists agreed (proportion agreement 89.1%) that the COVID-19 vaccine is rather unlikely to cause a disease flare [24]. This was somehow confirmed by a recent study that showed no difference on steroid use 30 days after vaccination between vaccinated and unvaccinated IBD patients [8]. No difference in clinical disease activity scores was also found in another cohort between anti-TNFa and non-anti-TNFa users after either vaccine dose irrespective of disease activity at vaccination (one third of each group reported not being in remission at vaccine administration [9]. Interestingly, in our cohort 6.4% and 8.4% of the patients reported novel gastrointestinal symptoms (predominantly diarrhea and abdominal pain) after D1 and D2 respectively. However, as the mean time between D2 and completion of the questionnaire was only 15 days, there is insufficient data to suggest that these symptoms reflected a true flare of disease rather than common mild AEs after any vaccination or simply an understandable anxiety status. 

In an attempt to find a possible correlation between the use of biologic agents and occurrence of AEs, we have proceeded to a sub-analysis according to treatment group (biologics, immunomodulators, combination therapy or no immunosuppression). The majority of studies emphasize that patients with immune-mediated inflammatory diseases receiving biologic therapies do not run any extra risk for AEs after COVID-19 vaccination [7,8,9,25,26] when compared with the general population. However, in the recently published PREVENT-COVID study anti-TNFa and vedolizumab use were associated with severe systemic reactions to D2 [10] while there is one report showing that the AE risk might even be reduced while on biologics [7]. The later can be partly explained by the fact that such agents (anti-TNFα) reduce the cytokine activation taking place after the inflammatory response to vaccination and therefore patients experience less AEs [3]. In fact, this somehow-protective anti-TNF effect has also been validated even in COVID-19 infection cases [27]. Whether this decrease in adverse event rates might reflect a reduced serologic response to vaccination is yet to be defined, but recently observed data do indicate an impaired antibody production after vaccination against COVID-19 in anti-TNFα recipients [9,28,29]. Similar negative correlation to AE occurrence was found for azathioprine in a cohort of 488 IBD patients [30]. In our study, no difference was found in the AEs risk between those under any kind of immunosuppression and those without such treatment. There was only an increased AE risk reported after D2 by patients receiving corticosteroids, but this observation could not be attributed exclusively to corticosteroids or to dual- or triple-combination immunosuppressive treatment due to the small sample size. 

There is a significant heterogeneity of immune reactions and AEs between the two basic vaccine formulations—mRNA and viral vector—administered in our study. A possible explanation could be their different manufacturing techniques [31]. We found that vaccine type is a risk factor for AE occurrence, but results are conflicting between the two vaccine doses (Table 3 and Table 5). As demonstrated in a meta-analysis of randomized controlled trials, mRNA vaccines are associated with higher AE rates [32] balancing with the higher efficacy rates they offer [33]. However, there are studies showing the opposite results as well [34,35].

To our knowledge, this is one of the first studies reporting on the perception and safety of vaccination against COVID-19 in a large European IBD population. It is a multicenter study, including patients that represent various areas of Greece. The limitations thereof are: (i) the subjective nature of the study design, since it was based on patient self-reporting that might lead to over- or under-estimation of some AEs; (ii) the potential recall bias for D1 AEs, as the questionnaire was completed once after vaccine completion; (iii) the predominance of mRNA vaccines; and (iv) the small sample size for sub-group analysis, especially for certain drugs administered for IBD (i.e., corticosteroids).

## 5. Conclusions

In conclusion, our study offers validation of the favorable safety profile of vaccines against COVID-19 in IBD patients. Patients can be reassured that they do not carry any extra risk for AEs occurrence compared with the general population, even though most of them receive immunosuppressive medications. In this way confidence can be gradually built and vaccine hesitancy can be diminished. In order to ensure vaccines’ long-term safety and efficacy, more studies, along with more prolonged post-marketing safety surveillance, are needed.

## Figures and Tables

**Table 1:** Demographic and clinical characteristics of inflammatory bowel disease patients included in the study (*N* = 1007).

Demographic and Clinical Characteristics		
gender (*N* (%))	male	509 (50.5%)
age (years; median (IQR))		44 (35–55)
disease (*N* (%))	crohn’s disease ulcerative colitis	648 (64.3%) 340 (33.7%)
disease duration (years; median (IQR))		10 (5–16)
current smokers (*N* (%))		323 (32%)
BMI (median (IQR))		25.99 (22.95–29.40)
blood type and rhesus factor (*N* (%))	A+/A−	284 (28.2%)/18 (1.8%)
	B+/B−	59 (5.9%)/8 (0.8%)
	AB+/AB−	50 (5%)/3 (0.3%)
	0+/0−	189 (18.8%)/46 (4.6%)
	unknown	350 (34.8%)
self-reported disease activity (*N* (%))	remission	832 (82.6%)
presence of co morbidities (*N* (%))		447 (44.4%)
treatment (*N* (%))	all biologics	647 (64.3%)
	anti-TNFa anti-α4β7 integrin anti-IL12/23	454 (45.1%) 77 (7.6%) 115 (11.4%)
	IMM monotherapy	131 (13%)
	no immune modifying therapies	213 (21.2%)
	systemic corticosteroids	32 (3.2%)
prior COVID-19 infection (*N* (%))		26 (2.6%)
type of vaccine (*N* (%))	BNT162b2 (Pfizer/BioNtech) mRNA-1273 (Moderna/NIH)	899 (89.3%) 18 (1.8%)
	ChAdOx1 (Astrazeneca) Ad26.CoV2.S (J&J)	79 (7.8%) 11 (1.1%)
other vaccinations during the last season (*N* (%))	influenza	797 (79.1%)
	streptococcus pneumoniae	222 (22%)
	other	29 (2.9%)
prior COVID-19 infection (*N* (%))		26 (2.6%)

BMI: body mass index; COVID-19: corona virus disease 19; IQR: interquartile range; IMM: immunomodulator; *N*: number; J&J: Johnson and Johnson.

**Table 2:** Adverse events (AEs) after vaccine dose 1 (D1) and dose 2 (D2) and statistical differences between all categories (positive answers/total answers).

AEs	D1	D2	D1 vs. D2
total AEs	81% (807/1002)	76% (727/956)	−5%, *p* = 0.0094
injection site symptoms	73% (730/998)	67% 642/952)	−6%, *p* = 0.0001
systemic AEs	44% (439/1002)	51% 489/956)	+7%, *p* < 0.0001
fatigue/malaise	35% (349/993)	43% (412/949)	+8%, *p* < 0.0001
allergic reaction	1% (10/989)	1% (9/943)	0%, *p* = NS
fever/chills	9% (89/986)	21% (195/950)	+12%, *p* < 0.0001
Lymphadenopathy	2% (19/987)	4% (39/943)	+2%, *p* = 0.001
joint/muscle pain	19% (190/984)	26% (249/945)	+7%, *p* < 0.0001
diarrheas (not pre-existing)	5% (47/990)	7% (64/943)	+2%, *p* = 0.007
abdominal pain (not pre-existing)	4% (42/988)	6% (57/945)	+2%, *p* = 0.035

**Table 3:** Determinants of vaccine systemic adverse events occurrence: results of the univariate analysis.

	Univariate Analysis			
	D1		D2	
	OR (95%CI)	*p*	OR (95%CI)	*p*
age (years) *	0.99 (0.97 to 0.99)	0.0003	0.97 (0.96 to 0.98)	<0.0001
gender (male vs female)	1.89 (1.47 to 2.42)	<0.0001	2.14 (1.66 to 2.77)	<0.0001
disease type (CD vs UC)	1.08 (0.86 to 1.35)	0.5	1.10 (0.87 to 1.38)	0.4
disease duration (years) *	1.00 (1.00 to 1.01)	0.4	1.00 (1.00 to 1.01)	0.5
Smoking	1.05 (0.80 to 1.37)	0.7	1.06 (0.81 to 1.40)	0.7
BMI *	0.99 (0.97 to 1.01)	0.2	0.96 (0.94 to 0.98)	0.0008
reported inactive disease	0.65 (0.46 to 0.91)	0.012	0.91 (0.64 to 1.30)	0.6
Comorbidities	1.09 (0.90 to 1.32)	0.4	0.88 (0.71 to 1.08)	0.2
anti-TNFa(infliximab, adalimumab, golimumab)	0.89 (0.69 to 1.14)	0.4	1.09 (0.84 to 1.40)	0.5
anti- α4β7 integrin (vedolizumab)	1.24 (0.77 to 1.98)	0.4	0.82 (0.50 to 1.33)	0.4
Anti-IL12/23(ustekimumab)	1.09 (0.73 to 1.60)	0.7	0.79 (0.53 to 1.17)	0.2
IMM monotherapy	0.85 (0.59 to 1.24)	0.4	1.12 (0.77 to 1.63)	0.5
Corticosteroids	2.52 (1.20 to 5.29)	0.1	2.45 (1.07 to 5.61)	0.035
no treatment	1.16 (0.86 to 1.57)	0.3	0.96 (0.70 to 1.31)	0.8
prior COVID-19 infection	1.78 (0.81 to 3.92)	0.1	3.13 (1.14 to 8.60)	0.03
type of vaccine (mRNA vs. vector)	2.81 (1.78 to 4.44)	<0.0001	0.26 (0.15 to 0.47)	0.0001

* analyzed as continuous variables, increasing values.

**Table 4:** Correlation of blood type and rhesus factor with systemic adverse events: results of the univariate analysis.

	D1		D2	
Blood Type	OR (95%CI)	*p*	OR (95%CI)	*p*
A+	0.97 (0.71 to 1.33)	0.8	1.03 (0.75 to 1.41)	0.8
A−	1.21 (0.47 to 3.09)	0.7	1.44 (0.55 to 3.77)	0.4
B+	0.47 (0.26 to 0.84)	0.01	0.68 (0.39 to 1.19)	0.1
B−	0.17 (0.02 to 1.39)	0.1	0.13 (0.02 to 1.04)	0.054
AB+	1.90 (1.05 to 3.41)	0.03	2.04 (1.08 to 3.85)	0.03
AB−	2.42 (0.28 to 26.77)	0.4	1.82 (0.16 to 20.19	0.6
0+	1.07 (0.76 to 1.50)	0.7	1.11 (0.78 to 1.57)	0.5
0−	1.22 (0.67 to 2.22)	0.5	0.58 (0.31 to 1.09)	0.09

**Table 5:** Determinants of vaccine systemic adverse events occurrence: results of the multiple regression analysis.

	Multivariate Analysis			
	D1		D2	
	OR (95%CI)	*P*	OR (95%CI)	*p*
Age	0.98 (0.97 to 0.99)	0.0001	0.97 (0.96 to 0.98)	<0.0001
female gender	1.94 (1.49 to 2.52)	<0.0001	2.15 (1.64 to 2.82)	<0.0001
BMI	-	-	0.98 (0.96 to 1.01)	0.1
blood type B+	0.42 (0.22 to 0.78)	0.005	-	-
blood type B−	0.17 (0.02 to 1.44)	0.08	0.11 (0.01 to 1.00)	0.025
blood type AB+	2.03 (1.08 to 3.81)	0.03	2.58 (1.30 to 5.12)	0.006
reported inactive disease	0.70 (0.49 to 0.99)	0.04	-	-
Corticosteroids	1.92 (0.88 to 4.19)	0.1	2.62 (1.07 to 6.43)	0.03
prior COVID infection	-	-	2.59 (0.92 to 7.29)	0.06
type of vaccine (mRNA vs. vector)	3.62 (2.22 to 5.92)	<0.0001	0.33 (0.18 to 0.60)	0.0002

## Data Availability

The data underlying this article will be shared on reasonable request to the corresponding author.
